# Water pollution and administrative division adjustments: A quasi-natural experiment in Chaohu Lake, China

**DOI:** 10.1371/journal.pone.0257067

**Published:** 2022-03-31

**Authors:** Jing Li, Di Liu, Mengyuan Cai

**Affiliations:** 1 School of Economics, Hefei University of Technology, Hefei, Anhui Province, People’s Republic of China; 2 Economics Division, School of Social Sciences, Nanyang Technological University, Singapore, Singapore; Shandong University of Science and Technology, CHINA

## Abstract

Administrative division adjustments, such as agglomerations, upgrading, and revocation, introduce a series of uncertain impacts on the social and economic development in administrative regions. Previous studies have focused more on the economic effects of administrative division adjustments, but in this paper, we also consider the environmental effects of such adjustments. In 2011, with the approval of the State Council, the prefecture-level Chaohu city was officially revoked, resulting in a county-level Chaohu city. One district and four counties under the jurisdiction of the original Chaohu city were placed under the jurisdiction of Hefei, Wuhu, and Ma’anshan. This adjustment made Chaohu Lake an inner lake of Hefei city. The administrative division adjustment of Chaohu Lake, China, is used as a quasi-natural experiment to explore the influence of such an adjustment on pollution control. The synthetic control method (SCM) is used in this study to evaluate the effect of the administrative division adjustment on the water quality indicators of Chaohu Lake. The following conclusions are drawn. First, after the administrative division adjustment, some water quality indicators, such as ammonia nitrogen, improved; however, other major pollution indicators, such as chemical oxygen demand (COD) and dissolved oxygen (DO), worsened to varying degrees. Second, the results reveal that improper development ideas, excessive industrial expansion, and the shift in economic growth and environmental goals were problems after the adjustment. Returning to the original intention of the administrative division adjustment, rationalizing the Chaohu Lake management system and designing a sound and feasible accountability mechanism are fundamental measures for reducing pollution.

## 1. Introduction

Administrative division adjustments are often used as an administrative tool to solve problems related to economic development or to stabilize the conflicts between a core city and its neighboring cities [1]. The fine-tuning of administrative areas can be used to solve regional problems and promote regional development. In Western countries, the administrative divisions adjustments are aimed to promote regional coordinated development, reduce administrative costs, and improve the bureaucracy’s efficiency [2, 3]. In contrast, conflicts in the administrative divisions of China reflect a misalignment between the political system and economic reforms [4] Since the reform and opening up in 1978, China’s administrative divisions have been constantly adjusted as part of the national strategy to promote rapid economic development [1], because China’s urban and regional development has been governed by territorial management in the transition from a planned economy to a socialist market economy [5]. Consequently, there are obvious characteristics of an "administration area-delimited economy" in China.

The environment is a typical public good. Public goods and public services are nonexclusive and noncompetitive, which can easily lead to conflicts of interest between different local governments in regard to environmental governance issues. Such conflicts result in serious resource waste, environmental pollution, and damage. In recent years, increasingly serious problems, such as explosive population growth, the excessive consumption of resources, environmental pollution, and ecological damage, have become major global issues [6], and environmental issues have received increasing attention. To cope with environmental governance issues in economic development, the 19th National Congress of the Communist Party of China proposed the "five in one" development concept and implemented the strictest environmental protection system. It is possible to try to solve environmental problems in administrative regions by adjusting the administrative divisions. However, due to its wide scope and complex content, research in this field is relatively limited, and its pertinence is relatively weak. Because of the numerous environmental factors and the difficulty of data acquisition, there are limited studies in this area, and no consistent conclusions have been drawn. Some studies believe that administrative division adjustments have a positive impact on environmental governance [7]. They used the geographical detector and evolutionary tree model to quantify the mechanisms and the effects of administrative division adjustments on changes in the PM2.5 concentration in three mega-urban agglomerations in China, i.e., the Beijing-Tianjin-Hebei region, the Yangtze River Delta, and the Pearl River Delta, during the 2000–2017 period. It found positive evidence for the effects of administrative division adjustments on pollution control. Some scholars hold the opposite view, arguing that if authorities use frequent administrative division and administrative system adjustments to alleviate the contradiction between the economic foundation and the superstructure, they cannot establish a good regional governance model [4] and cannot fundamentally eliminate the economic problems in an administrative region. Even worse, such adjustments may instead be a legitimate reason for land expansion, resulting in a failure of environmental governance [8].

Administrative division adjustment is an inherent requirement of economic and social development. The existing administrative division in Anhui Province are formed based on historical administrative division. Although they have been adjusted several times, the scales of the central cities are small, and the size of the administrative districts vary. Chaohu Lake is the fifth largest freshwater lake in China and the largest lake in Anhui Province. Its water area covers approximately 825 square kilometers. Chaohu Lake is not only an important channel connecting the Jianghuai area to the north but also an important agricultural product base. The Chaohu Lake basin covers a total area of 13,350 square kilometers. The management of the water system, especially Chaohu Lake is not efficient, which has formed a great restriction on the current stage of economic and social development. Before the administrative division adjustment, it spanned 11 cities and counties, including Hefei, Chaohu, and Lu’an. Due to rapid social and economic development, the water quality of Chaohu Lake has gradually deteriorated. The problem of pollution in Chaohu Lake has attracted the attention of the central and local governments. According to the *Outline of the 12th Five-Year Plan for Water Pollution Prevention and Control in Chaohu Lake* reported by the *Ministry of Ecology and Environment of the People’s Republic of China*, during the 11^th^ Five-Year Plan (2006–2010) (https://www.mee.gov.cn/gkml/hbb/bgt/201012/W020101206595119451462.pdf), a total of 669 million yuan was obtained from the central government’s transfer payment funds and the central budget for infrastructure and treasury bonds, accounting for 9.5% of the total planned investment in the 11th Five-Year Plan project. However, the pollution situation of Chaohu Lake has not been greatly improved. During the *National Two Sessions* in 2010, the Provincial People’s Congress delegation put forward the *Proposal on Scientific and Timely Administrative Divisions Adjustment to Meet the Needs of Economic Development*, which was included in the key motion of the National People’s Congress. Before 2012, Chaohu Lake was jointly governed by Hefei and the prefecture-level Chaohu city. There was a lack of effective coordination between them. Thus, pollution was not significantly alleviated, which seriously restricted the regional economic development of the Chaohu Lake basin and affected the production and life of local residents [9]. Pollution control involved many departments, such as industry, agriculture, and environmental protection. To a certain extent, there were frequent excuses and wrangling in the governance of Chaohu Lake, and many good measures would eventually have to be implemented in a compromised manner after being coordinated. The external diseconomy of water pollution was instantly magnified due to the administrative division. In July 2010, the National People’s Congress and the Ministry of Civil Affairs organized a joint research team to conduct a survey on the administrative division adjustment. Based on full investigation and demonstration, the provincial government formally reported to the State Council the *Request for Instructions on the Revocation of Chaohu City and the Adjustment of Relevant Administrative Divisions*. On August 22, 2011, with the approval of the State Council, the administrative division of Chaohu city was adjusted. The prefecture-level Chaohu city was officially revoked, resulting in a county-level Chaohu city. One district and four counties under the jurisdiction of the original Chaohu city were placed under the jurisdiction of Hefei, Wuhu, and Ma’anshan. This adjustment made Chaohu Lake an inner lake of Hefei city, which facilitated the overall management of Chaohu Lake and provided development opportunities for Hefei. The effect of the administrative division adjustment on the treatment of Chaohu Lake is considered in a quasi-natural experiment in this study. In the literature, there is a popular trend of regarding place-based policies in China as quasi-experiments and analyzing their effects on pollution. For example, Yu and Zhang [10] regarded the implementation of the smart city policy as a quasi-natural experiment and evaluated its effect on energy efficiency. Similarly, She et al. [11] used the difference-in-differences (DID) method to identify the effect of River Chief Policy (RCP) on surface water pollution. They claimed that the RCP provides a quasi-experimental environment in which the cities that implemented the RCP can be defined as having RCP status. Likewise, the Two Control Zone policy [12], New Energy Demonstration City policy [13], and coal-to-gas policy [14] are all considered quasi-natural experiments when considering pollution outcomes. Although it is inevitable that the implement of these place-based policies is not totally random because of confounding factors, literature relies on a variety of checks, such as parallel trend test and placebo test, to examine the validity and robustness of the causal inferences.

In this paper, the administrative division adjustment of Chaohu Lake is used as a quasi-natural experiment to explore the influence of the adjustment on pollution control. Using data from the Ministry of Environmental Protection, we choose the synthetic control method (SCM) commonly used in project evaluation to identify the effect of the administrative division adjustment on the water quality indicators of Chaohu Lake and to discuss the internal mechanism of this effect. Our study contributes to the literature on the environmental impacts of administrative division adjustments and holds great significance for understanding the environmental effects of administrative divisions and grasping the connotations of high-quality development.

## 2. Adjustment of the Chaohu administrative division and pollution in Chaohu Lake

The original district-level Chaohu city consisted of one district and four counties. The city scale was small, and the level of economic development was relatively poor. In addition, the differences among political districts were large, and problems related to river governance were severe. In particular, the water management system was not effective; hence, the pollution regulation of Chaohu Lake did not fundamentally improve. To promote social and economic development and to rationalize the management system for Chaohu Lake, in 2011, one district and four counties of the prefecture-level city were divided into three cities. Lujiang was incorporated into Hefei; Hanshan and Hexian were incorporated into Ma’anshan; and Wuwei was incorporated into Wuhu.

China’s environmental protection system follows "territorial management and hierarchical responsibility". The principle of territorial management requires relevant departments to manage the environmental issues under their jurisdiction, which can strengthen the rights and responsibilities of local environmental protection departments in regard to environmental supervision. However, the territoriality principle has disadvantages for rivers and lakes that cross administrative regions. Each department is responsible only for the effectiveness of lake management in its own region, which makes it difficult to implement comprehensive lake planning. Before the abolition of the prefecture-level municipality of Chaohu, Chaohu Lake was jointly managed by Hefei city and Chaohu city and was divided into east and west lakes for management. As a result, all departments considered development and utilization planning only for their portion of the lake, and most administrative regulations and methods could not be effectively implemented. In such scenarios, disputes caused by regional joint governance are inevitable, and pollution control is difficult to achieve. After the abolition of the prefecture-level Chaohu city, Chaohu Lake was under the independent jurisdiction of Hefei city, which avoided regional disputes caused by the joint governance of different administrative regions. Therefore, the abolition of the prefecture-level Chaohu city was conducive for Hefei to formulate independent overall planning and to clarify the responsibilities of government departments at all levels. In addition, compared with the prefecture-level Chaohu city, Hefei city has a developed economy, perfect urban functions, complete infrastructures, and a strong economic background. As a result, Hefei is able to analyze and study the environmental carrying capacity of Chaohu Lake more scientifically, providing the possibility for better pollution control measures for Chaohu Lake.

In addition, the government has made efforts to control Chaohu Lake. Table 1 shows that during different five-year planning periods, the government invested considerable money to address the problem of pollution in Chaohu Lake. At the beginning of 9^th^ Five-Year Plan (1996–200), the water quality of Chaohu Lake was worse than Class V water quality standard. The target of this phase was to reach the Class IV, while over 30% river did not reach that standard. In 2009, among the 10 main rivers in the Chaohu Lake basin, Yuxi River was the only river whose water quality reached the Class III standard. There are 9 rivers that have not reached the standard, of which: Shiwuli River, Nanfeihe River, Paihe River and Shuangqiao River are inferior to Category V (the planning target is Class V), with the COD, ammonia nitrogen, and total phosphorus exceeding the standard; Zhaohe, Zhagao River, Baishitian River, Hangbu River and Fengle River are in Class IV (the planning target is Class III), with the petroleum exceeding the standard. Before the administrative division adjustment (year 2011), pollution control governance was in progress without significant outcomes. However, after 2011, water quality indicators, such as NH3N, improved significantly, while other major pollution indicators, such as COD and DO, worsened to varying degrees because of rapid industrial and urban development. Recently, according to the Chaohu Basin Water Pollution Prevention Regulations revised in December 2019, water quality of Chaohu Lake and Fengle River, Hangbu River, Baishitian River, Zhaohe, Zhagao River, Yuxi River, and Pai River shall reach the Class III water quality standard, and the Nanfei River and Shiwuli River shall reach the Class IV.

**Table 1:** Number of governance funds and projects invested in Chaohu Lake, 1996–2015.

Period	Fund (Billion Yuan)	Number of Completed Projects	Pollution Situation
Ninth Five-Year Plan (1996–2000)	2.58	3000+	The progress of industrial wastewater treatment was slow, and the water pollution control did not reach the target.
Tenth Five-Year Plan (2001–2005)	3.03	26	Pollutant emissions did not meet planned targets.
Eleventh Five-Year Plan (2006–2010)	7.07	56	Due to the need of the Chaohu Lake Basin to undertake the demonstration zone of industrial transfer in the Wanjiang Urban Belt, the amount of industrial wastewater and pollutants also increased by more than 30%, and the pressure on environment risk prevention increased.
Twelfth Five-Year Plan (2011–2015)	10.9	117	Water quality in Chaohu Lake has been improved, but overall eutrophication status has not fundamentally changed.

Note: The data were extracted from *the 10*^*th*^*-12*^*th*^ *Five-Year Plan for Water Pollution Prevention and Control in the Chaohu Lake Basin* and *The Overall Plan for Water Environment Treatment of Chaohu Lake*, *Anhui Province*.

After the abolition of the prefecture-level municipality of Chaohu, Hefei gained geographical space for development and paid more attention to economic development: The governance of Chaohu Lake was focused on assisting industrial development. The rapid development of Hefei in recent years has led to a sharp increase in the scale of river basin construction and rapid population growth, and the continuous accumulation of pollution in production and life has far exceeded the carrying capacity of Chaohu Lake itself. Although the causes of pollution are obvious, they have not been taken into account in the process of development and construction around Chaohu Lake in recent years. Environmental supervision has noted that in the context of the rapid economic and social development of the Chaohu Lake basin, the water pollution in Chaohu Lake has shown an improving trend, but the "Regulations on the Prevention and Control of Water Pollution in the Chaohu Lake Basin" has not been successfully implemented (http://www.gov.cn/hudong/2017-07/30/content_5214708.html). The division of the first, second, and third protection areas of Chaohu Lake is one of the core provisions of the most stringent regulation in history. However, this work remains in the stage of expressing basic principles when it should have become the safety bottom line of Chaohu Lake protection, and it has been put on the shelf. Whether the regulations can be enforced is essentially a question of who gives way when there is a conflict between environmental protection and development: In Chaohu Lake, pollution control frequently gives way to economic development.

Table 2 shows that the eutrophication status of the western half of Chaohu Lake is generally worse than that of the eastern half. From the perspective of the comprehensive eutrophication state index, the data do not change substantially, and overall, they do not show a positive trend. Although Hefei has invested considerable amounts of money, the existing Chaohu Lake governance focuses on road construction, river regulation, and comprehensive ecological measures. The eutrophication status of Chaohu Lake has not fundamentally improved. In recent years, with the high incidence of water blooms, the cyanobacteria data reported by the environmental supervision group highlight the urgent need for pollution control. According to reports from the Ministry of Ecology and Environment of the People’s Republic of China, in 2015, the largest bloom area was 321.8 square kilometers, accounting for 42.2% of the whole lake, the highest percentage in nearly eight years. In contrast, it was 237.6 square kilometers in 2016, accounting for 31.2% of the entire lake.

**Table 2:** Eutrophication status of Chaohu Lake from 2009 to 2016.

Year	Comprehensive Eutrophication State Index			Eutrophication Status		
	Eastern half	Western half	Whole	Eastern half	Western half	Whole
2009	52.3	64.8	60.1	LE	SE	ME
2010	51.0	64.2	59.0	LE	ME	LE
2011	52.3	63.7	59.5	LE	SE	ME
2012	53.5	60.9	57.4	LE	ME	LE
2013	52.2	60.0	55.8	LE	ME	LE
2014	50.5	63.1	57.1	LE	SE	ME
2015	50.6	62.8	57.2	LE	ME	LE
2016	50.1	62.5	56.8	LE	ME	LE

Note: LC: light eutrophication; ME: medium eutrophication; SE: serious eutrophication.

The data were extracted from the “China Environment Yearbook 2010–2017” and the National Surface Water Monitoring Station of the Ministry of Environmental Protection.

## 3. Literature review

In general, several significant impacts are considered in the assessment of administrative division adjustments. The most common effects are economic effects, which are the focus of many previous studies, while a small number of studies evaluate the environmental effects of administrative division adjustment. A brief review of the literature on these two major effects is included in the following discussion.

### 3.1 Review of the literature on the economic effects of administrative division adjustments

Some studies have examined the impacts of administrative division adjustments on local economic development in terms of social and political systems and economic development stages. For instance, Wagenaar et al. [15] found significant correlations between administrative division adjustments and local economic growth in the US. Ideally, administrative division adjustments can lead to substantial changes in local economic development. In the US, administrative division adjustments are often implemented to control the spread of diseases and to protect the local ecology, and they are rarely implemented for purposes of economic development. A wave of city mergers to form metropolises in Canada during the 1990s primarily aimed to reduce administrative costs and improve the efficiency of public services [16]. The mergers overcame the barriers of administrative boundaries to integrated local development, especially declines in core cities, and they generated remarkable positive effects. Redding et al. [17] considered the German division and reunification as a natural experiment and studied whether strong correlations exist between the local economic development of border cities and administrative division adjustments. Similarly, Abadie et al. [18] assessed the impacts of reunification on the per capita gross domestic product (GDP) of West Germany. They found that the impacts of administrative division adjustments due to German reunification emerged in 1992 and that East Germany was a burden on West Germany’s economic development.

Due to the special features of China’s economic system, administrative division adjustments are often implemented for economic purposes. Administrative division adjustments are a major guiding factor of urbanization in China and have far-reaching economic and social impacts on local development [19]. A vast literature on this topic exists, and the extensive studies on administrative division adjustments in China generally fall into two groups. The first group is based on case studies of administrative division adjustments in a specific region (province/municipality), and the studies in this group compare the local economic volume and economic growth before and after the adjustments. For instance, Zhao [20] studied administrative division adjustments in Sichuan Province between 1993 and 1998. The results reveal that the administrative division adjustments did indeed boost Sichuan’s economic growth. The second group analyzes the causes, patterns, impacts, and reform approaches of administrative division adjustments in China. The historical establishment of administrative divisions is based on national unity and political stability, and modern administrative division adjustments are implemented mainly to address the evolving needs of rapid economic and social development and urbanization [21, 22]. Yu et al. [23] provided a comprehensive summary of administrative division adjustments at the province and prefecture levels in China. They concluded that administrative division adjustments have been a major factor influencing the progress of urbanization and that they have been a substantial driving force for the evolution of the urban spatial structure in China.

In general, the existing literature on how administrative division adjustments affect local economic development can be classified into two categories. The first category focuses on the overall influences of administrative division adjustments on local economic development. Due to variations in the development levels of different regions and different administrative division adjustment approaches, adjustments are implemented in different ways. Moreover, the effects of adjustments on economic development are not always positive and vary from region to region. The other category focuses on analyzing how administrative division adjustments affect a specific aspect of the local economy, such as industrial upgrading, urbanization, and interregional inequality. More studies fall into this category, with more specific analytical indicators, and the results vary greatly from study to study.

### 3.2 Review of the environmental effects of administrative division adjustments

A plethora of scholars have conducted studies on the impacts of administrative division adjustments on environmental governance. Most existing studies have considered economic impacts to be the primary study subject and covered changes in environmental aspects only in passing, or they have included a few qualitative assessments of the environmental impacts of such adjustments: Empirical assessments are very rare. There are rich studies focus on the transboundary transfer of pollution. The environmental standards and policies of different administrative divisions can cause a spatial reallocation of pollution, as lax environmental regulations can be effective in attracting current capital, and emission reduction can deter international investment [24, 25]. Consequently, polluting enterprises move their operations from one administrative division to another to avoid pollution control requirements. Cai et al. [26] discovered that polluting enterprises often prefer moving to administrative borders. Therefore, the shifting of pollution among different administrative boundaries is a major barrier to eliminating environmental pollution. Administrative division adjustments are important instruments for addressing environmental issues. Due to different social and political systems and economic development stages, there are fewer studies on administrative division adjustments outside China, and the adjustments are implemented mainly to protect ecological systems and to prevent the spread of disease.

Water is a core resource in sustainable development, and it is critical for socioeconomic development, ecological systems, and human survival [27]. Water resources involve many complex factors and correlations, including some interrelated natural, social, and economic elements. In the process of water resource utilization, complicated interrelations subject water resources to the influences of many uncertainties based on multiple factors [28]. Chinese local governments can exert administrative power only within their administrative divisions; the transboundary feature of pollution makes it impossible to hold a specific local government accountable [29]. The increasing competition and conflicts among different regions and sectors for water use enhance the significance of rational water allocation and utilization [30, 31].

To ensure that local governments comply with the environmental targets set in the 11th Five-Year Plan (2006–2010) released in 2005, the central government revised the performance assessment criteria for local officials, making performance in achieving pollution control targets a key indicator in determining the career development of local officials [29]. Local officials who failed to achieve pollution control targets could be fired [32]. The new system gave local officials a strong incentive to achieve national environmental targets [33]. Chinese scholars have not conducted many studies on the environmental governance effects of administrative division adjustments. Instead, they have tended to conduct simple analyses of environmental governance impacts while studying the overall impacts of administrative division adjustments, or they have employed administrative division adjustments as one of the factors influencing the changes in a specific region’s ecological and environmental governance during a specific period.

## 4. Methods

To investigate how the abolition of the prefecture-level municipality of Chaohu could affect water quality in Chaohu Lake in the Hefei region, analysis would typically have to be performed under a counterfactual framework. One popular approach to identifying the causal effects of certain events is the DID method [34]. The DID design is a quasi-natural experimental setting in which randomized controlled trials (RCTs) are infeasible. Nonetheless, causal interpretation of DID results relies on a set of important assumptions [35]. One of the most important assumptions is the parallel trend assumption, which requires that the control group and the treatment group have similar trends in terms of the key variables of interest before the treatment. However, the factors determining environmental governance vary in different cities, and the factors affecting the water quality of rivers and lakes are complicated even without administrative division adjustments. Therefore, the hypothesis of convergence among individual samples under the DID approach cannot be satisfied. The matching method does not work neither, as it is impossible to find a region similar to the Hefei region in all aspects that did not undergo an administrative division adjustment. Moreover, in terms of geographical, historical, regional, and other factors, different lakes have different levels of water pollution, and it is impossible to find a lake that is similar to Chaohu Lake.

For these reasons, this paper adopts the SCM proposed by Abadie et al. [36] to identify the causal effect of the administrative division adjustment of Chaohu Lake. This approach is commonly used in project evaluation and is powerful in identifying the causal effects of a specific event [37–39]. For example, Chen et al. [37] used the SCM to study the effect of China’s sulfur dioxide emissions trading pilot scheme (SETPS) on industrial sulfur dioxide emissions. They found that the impact of the SETPS has regional heterogeneity. Tianjin’s emissions trading scheme showed the ideal reduction effect. However, the execution of the SETPS in different regions did not lessen SO_2_ emissions. In addition to environmental economics, the SCM is well adopted in health economics. Alfano et al. [38] found that provinces with open schools had more COVID-19 cases than their counterfactual level evaluated by the SCM, which suggests that opening schools caused an increase in COVID-19 cases. Another study of the impacts of COVID-19 was conducted by Xin et al. [39]. They used the SCM to evaluate the impacts of the COVID-19 pandemic on public transit. They found a reduction effect of the pandemic on ridership in most Chinese cities.

The basic idea of the SCM is as follows. To assess the effects of an event, Rubin’s counterfactual framework is established first to represent how an area would be in the absence of a specific event (counterfactual); then, the data under the counterfactual scenario are compared with the actual data after the event occurred. The differences between the two scenarios are known as treatment effects. Constructing data for a control group that is similar to the region is an important exercise. To create a control group using the SCM, the comparison is not made with one or multiple similar regions; instead, a reasonable control group is created based on a weighted average of all similar regions. The property variables for the control group are almost identical to those for the treatment group before the event and represent the status of the region in the absence of the event. The SCM has three major advantages in establishing a counterfactual framework. First, when designing the control group by calculating the weighted average of similar regions, the weights are decided by the data, not by subjective choices; each weight represents the region’s contribution to the control group. Second, the sum of the weights of all samples is equal to one, and such weights avoid excessive extrapolation. Third, the SCM requires each component region of the control group to be similar to the treatment region. For this reason, highly different regions should be excluded from the control group [40].

The study considers the abolition of the prefecture-level municipality of Chaohu as a treatment event and assesses its effects on the water quality of Chaohu Lake in the Hefei region. Assume time *t* ∈ [1, *T*], and let the year of administrative division adjustment be *T*_0_ $Y_{it}^{I}$ and $Y_{it}^{N}$ indicate that region *i* was affected and was not affected by the administrative division adjustment at time *t*. Then, $\alpha_{it}=Y_{it}^{I}-Y_{it}^{N}$ can represent the effects of the administrative division adjustment. In the case of the Hefei region, when *T*_0_ < *t* ≤ *T*, i.e., after the abolition of the prefecture-level municipality of Chaohu, $\alpha_{it}=Y_{it}^{I}-Y_{it}^{N}=Y_{it}-Y_{it}^{N}$, where *Y*_*it*_ represents the actual situation in Hefei, while $Y_{it}^{N}$ is the counterfactual value, i.e., what the situation in Hefei would have been in the absence of the administrative division adjustment.

To observe the administrative division adjustment of the municipality of Chaohu, a total of *J* + 1 samples are distributed into the treatment group (Hefei region) and control group. The factor model proposed by Abadie et al. [35] is used to synthesize $Y_{it}^{N}$, and the weights for the control group are defined as *w* = (*w*_2_, *w*_3_, …, *w*_*J*+1_)′, where *w*_*J*_ is the weight of synthetic control city *J*. For any given *w*_*J*_, the synthetic control city can be calculated as follows:

∑J=2J+1 wJYitN=σt+θt∑J=2J+1 wJZi+λt′∑J=2J+1 wJμi+∑J=2J+1 wJεit (1)

Where *σ*_*t*_ represents the time-independent effect of the abolition of the prefecture-level municipality of Chaohu, *Z*_*i*_ is the observed vector, *θ*_*t*_ is the unknown coefficient, $\lambda_{t}^{\prime }$ and *μ*_*i*_ are the unobservable interactive fixed effects, $\lambda_{t}^{\prime }$ represents common unobservable factors, *μ*_*i*_ is the unobservable regional fixed effects, and *ε*_*it*_ is the random error term.

When the intervention period *T*_0_ is close to infinite, the optimal w* should exist, and the synthetic control estimator is asymptotically unbiased. However, such conditions do not occur in reality. Usually, the unobservable characteristics of the synthetic control group are made similar to those of the treatment group. Abadie et al. [35] demonstrated that when *t* ≤ *T*_0_, if *w* enabling both $Z_{1}\approx \sum_{J=2}^{J+1}\;w_{J}Z_{i}$ and $Y_{it}\approx \sum_{ȷ=2}^{J+1}\;w_{J}Y_{it}^{N}$ can be found, then it could also lead to $\mu_{1}\approx \sum_{J=2}^{J+1}\;w_{J}\mu_{i}$. Therefore, the evolutionary pathway of the effect of the synthetic Hefei region is similar to the actual growth pathway of Hefei before the abolition of the municipality of Chaohu. After calculating the weight matrix using data from before the administrative division adjustment, $\sum_{J=2}^{J+1}\;w_{J}Y_{it}^{N}$ can be used as an unbiased estimator of $Y_{it}^{N}$. Hence, an unbiased estimate of *α*_*it*_ can be obtained as follows:

α^it=YitI-YitN=Yit-∑J=2J+1 wJYitN (2)

where ${\hat{\alpha }}_{it}$ represents the environmental and economic effects of the abolition of the prefecture-level municipality of Chaohu, *α*_*it*_ > 0 indicates that the effects of the administrative division adjustment are positive, and *α*_*it*_ < 0 indicates that the effects are negative.

## 5. Empirical results and analysis

The results of the synthetic control of the environmental effects on Chaohu Lake are discussed in this section, followed by several robustness tests.

### 5.1 Results of the synthetic control of the environmental effects on Chaohu Lake

In the SCM estimation, Hefei is used as the treatment group, and the remaining 69 samples are selected as the synthetic Hefei control group. The weights of 69 sample points are mostly nonzero, which ensures a comprehensive adoption of the samples. Moreover, samples that are more similar to Hefei are assigned larger weights. The chemical oxygen demand (COD), ammonia nitrogen (NH_3_N), and dissolved oxygen (DO) indicators are used to construct Hefei and synthetic Hefei’s indicator paths and their predictors. COD reflects the degree of organic pollution in water, and a higher COD is regarded as more serious pollution. NH_3_N is derived from synthetic chemical fertilizers and is usually related to agricultural emissions. Additionally, DO reflects water pollution, especially organic pollution. The variables of the study can be divided into two main categories, environmental and economic data, as shown in Table 3. The adoption of control variables follows Zhou et al. [41] and Wang [42]. In addition, we choose COD and NH_3_N as the measures of pollution because they are listed as key pollutants that are at the core of wastewater pollution management in the 11th-13th Five-Year Plans (2006–2020) and because Fujii and Managi [43] argued that COD and NH_3_N are the best indicators for assessing the degree of water pollution due to their better measurement methods.

**Table 3:** Descriptive statistics.

Variable	Descriptions	Obs	Mean	Std. Dev.	Min	Max
*Codmn*	Chemical Oxygen Demand (COD)	910	4.9255	9.2121	0.8412	132.4935
*Do*	Dissolved Oxygen (DO)	910	7.6759	1.7128	0.3622	12.9021
*nh*_*3*_ *n*	Ammonia Nitrogen (NH_3_N)	910	0.8258	1.9252	0.0276	24.1183
*Nph*	Abs(pH-7)	910	0.7054	0.3951	0.0031	2.0786
*Lny*	Logarithm of Cities’ GDP	910	6.8041	1.0909	3.6720	9.6460
*Lnpy*	Logarithm of GDP per Capita	910	10.1572	0.7911	8.0356	12.0288
*Urate*	Urbanization Rate (%)	910	53.4747	15.7986	19.9961	94.6300
*Lnpd*	Logarithm of Population Density	910	5.9325	0.7753	3.0540	7.0582
*Industry*	Degree of Industrialization (%)	910	43.3447	11.7010	11.0108	83.7707
*Lnagr*	Logarithm of the GDP of the Primary Industry	910	4.8177	0.9428	0.9973	6.3938
*lny* ^*2*^	Square of the Logarithm of GDP per Capita	3080	6.0416	1.0785	1.2141	8.8829
*Second*	Logarithm of the GDP of the Secondary Industry	3080	49.4137	10.8866	9.0000	90.9700
*Third*	Proportion of the Tertiary Industry in GDP (%)	3080	36.3191	8.4494	8.5800	85.3400
*Lnl*	Logarithm of Labor	3080	5.2643	0.7119	2.3627	7.4427
*Labpercent*	Proportion of Labor	3080	57.6016	9.7829	17.4469	95.4582
*Lnpop*	Logarithm of Population	3080	5.8312	0.6653	2.9014	8.0117
*Lnfix*	Logarithm of Fixed Asset Investment	3080	6.1287	1.0097	3.3841	9.4089
*Lnarea*	Logarithm of the Urban Area	3080	9.3550	0.8213	7.0148	12.4426

The environmental data are extracted from the national surface water monitoring station of the Ministry of Environmental Protection. The annual average data are reorganized and matched with the economic and social data of the city where the monitoring point is located. Because the SCM requires completely balanced panel data, some missing samples are deleted, and finally, a panel dataset of 70 samples covers four types of water quality data and six types of socioeconomic indicators. The economic data are mainly compiled from the China City Statistical Yearbook and Provincial Statistical Yearbook. We ensure that the synthetic control group is sufficiently close to the characteristics of synthetic Hefei. Tibet and some prefecture-level city data are excluded from the study. Table 3 shows the descriptive statistics of the environmental and economic variables.

**Fig 1:**
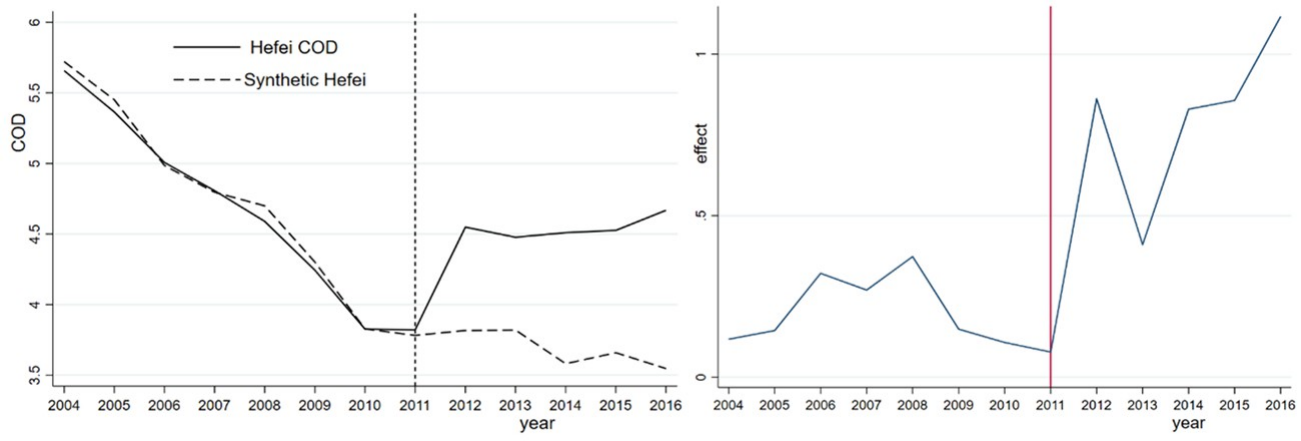
Hefei synthetic COD and its effect.

Fig 1 shows that prior to the abolition of the prefecture-level municipality of Chaohu in 2011, the COD indices of Hefei and synthetic Hefei coincide to a high degree, indicating that synthetic Hefei is a good counterfactual. After the abolition of the prefecture-level municipality of Chaohu, the COD indices of Hefei and synthetic Hefei display different trends. The COD index of synthetic Hefei continues to exhibit a downward trend, while that of Hefei shows an upward trend. This difference is mainly due to a series of rapid urban construction and industrial development projects implemented by the newly formed Binhu New District based on Chaohu city and Lujiang county, leading to a large amount of organic pollution in Chaohu Lake, which appears as a sharp rise in the COD index.

The annual COD difference between Hefei and synthetic Hefei is calculated to assess the effect of the administrative division adjustment on COD in Hefei. Before the adjustment, the COD gap between Hefei and synthetic Hefei is very small, but it increases after the abolition of the prefecture-level municipality of Chaohu in 2011. On the one hand, the COD index of synthetic Hefei matches that of Hefei; on the other hand, the adjustment of Chaohu city causes a higher COD level in Hefei. Table 4 compares the predictions of the COD index for Hefei and synthetic Hefei before the adjustment of the prefecture-level Chaohu urban area in 2011. From Table 4, we observe that GDP, GDP per capita, the value-added of the secondary industry, the urbanization rate, and population density are similar between the treatment group and the synthetic group.

**Table 4:** Treatment and synthetic control groups of the predictive variables for COD.

Predict Variables	Treated	Synthetic
*Lny*	6.8616	6.8819
*Lnpy*	9.9063	9.9362
*Urate*	60.0815	59.8289
*Lnpd*	6.5132	6.2169
*Industry*	38.3829	38.4740
*Lnagr*	4.7001	4.7139

Note: “Treated” represents the treatment group, and “Synthetic” represents the synthetic control group.

**Fig 2:**
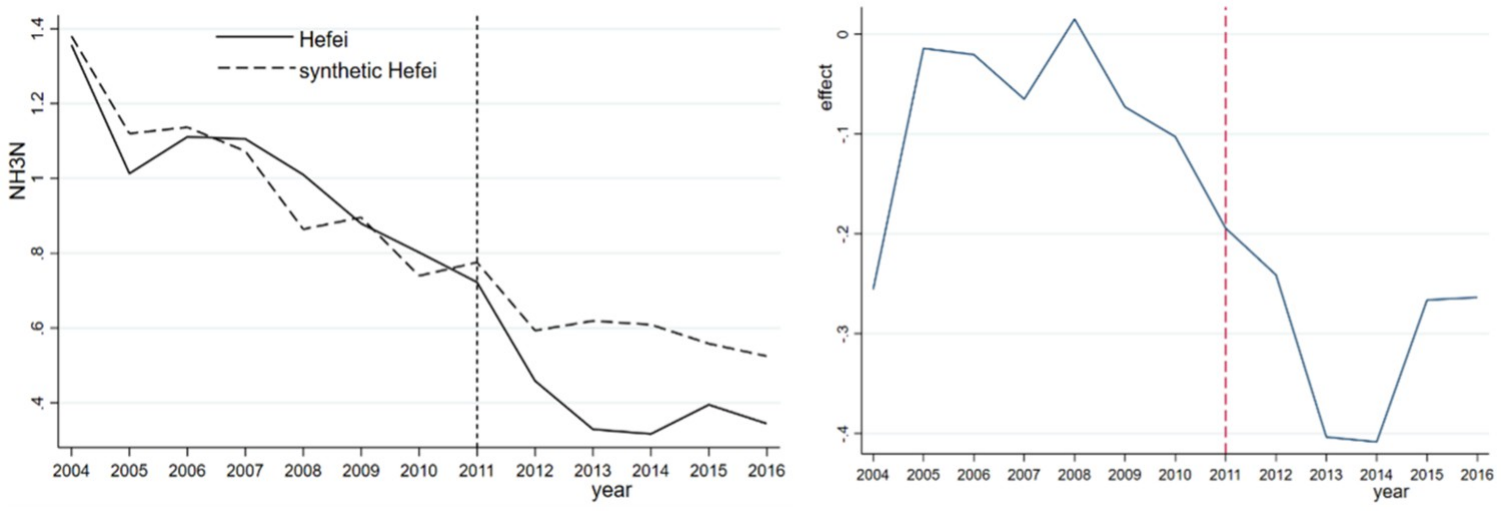
NH_3_N and its effect for Hefei and synthetic Hefei.

In contrast, the synthesized NH_3_N shows a steady downward trend, but the actual NH_3_N index declines sharply after the administrative division adjustment, implying a significant improvement in water quality. As previously noted, NH_3_N originates mainly from agricultural emissions rather than industrial emissions and is the result of rapid urban development in Hefei, which reduces the amount of land surrounding Chaohu Lake used for agricultural purposes. As a result, the use of chemical fertilizers and pesticides has declined significantly, directly reducing the amount of pollutants. From Fig 2, we observe a large gap between the NH_3_N indices of Hefei and synthetic Hefei during the 2011–2013 period. However, the gap gradually narrows, and the continuous improvement in NH_3_N in Hefei is not obvious after 2013. As reported in Table 5, the fitting error of each variable between the treatment group and synthetic control group is relatively small, that is, less than 0.1. In other words, synthetic Hefei is a good counterfactual for Hefei in terms of the ammonia nitrogen index.

**Table 5:** Treatment and synthetic control groups of the predictive variables for NH_3_N.

Predict Variables	Treated	Synthetic
*Lny*	6.8616	6.8502
*Lnpy*	9.9063	9.8948
*Urate*	60.0815	60.0211
*Lnpd*	6.5132	6.5062
*Industry*	38.3829	38.3609
*Lnagr*	4.7001	4.6916

Note: “Treated” represents the treatment group, and “Synthetic” represents the synthetic control group.

**Fig 3:**
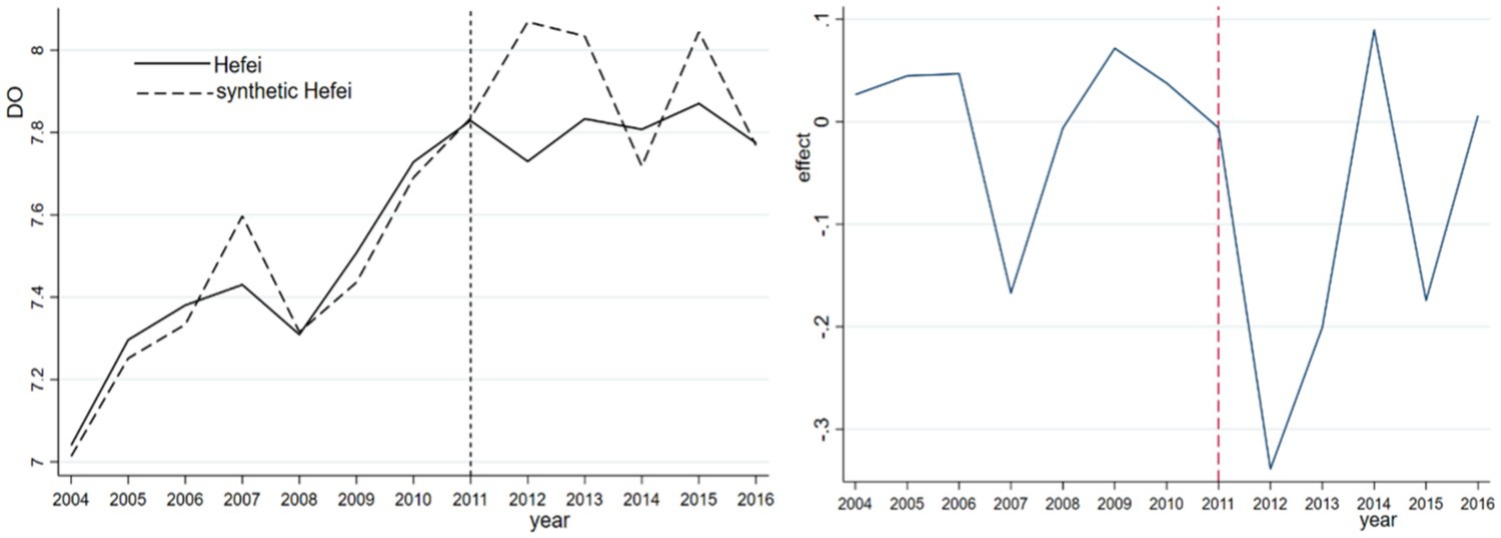
DO and its effect for Hefei and synthetic Hefei.

In terms of the DO indices, as illustrated in Fig 3, similar levels of DO are observed for Hefei and synthetic Hefei prior to 2011. Although the synthetic DO index increases annually from 2011 to 2016, there is no obvious improvement in the DO index in Hefei. Similar to COD, the DO index is not improved due to the vigorous development of the shores of Chaohu Lake and the construction of a large number of buildings without corresponding environmental protection measures. Unsurprisingly, the DO index of Hefei is always lower than that of synthetic Hefei, especially one year after the adjustment. Table 6 compares the predicted DO levels of Hefei and synthetic Hefei before the abolition of the prefecture-level municipality of Chaohu in 2011. The regression results for each variable indicate that the difference between Hefei and synthetic Hefei is very small, indicating that a comparison of the fitting effect is appropriate.

**Table 6:** Treatment and synthetic control groups of the predictive variables for DO.

Predict Variables	Treated	Synthetic
*Lny*	6.8616	6.8491
*Lnpy*	9.9063	9.8864
*Urate*	60.0815	60.0184
*Lnpd*	6.5132	6.4997
*Industry*	38.3829	38.2643
*Lnagr*	4.7001	4.6896

Note: “Treated” represents the treatment group, and “Synthetic” represents the synthetic control group.

### 5.2 Robustness checks

To validate the results, several robustness checks are conducted in this study. First, the DID method is the most intuitive method in policy evaluation and can be used to compare the changes in the water quality of Chaohu Lake after the administrative division adjustment and the changes in water quality at other monitoring points. Table 7 shows the regression results using the DID approach.

**Table 7:** DID method regression results.

Variables	COD	NH_3_N	DO
*chao×t*	-7.878**	-3.347***	1.015
	(3.747)	(0.683)	(0.980)
*Lny*	11.04***	3.341***	-3.055***
	(3.801)	(0.645)	(0.780)
*Lnpy*	-3.352	-1.501***	3.185***
	(2.790)	(0.486)	(0.697)
*Urate*	-0.267***	-0.0224**	-0.0226
	(0.0892)	(0.0108)	(0.0180)
*Lnpd*	7.040*	-0.185	-0.998
	(4.250)	(0.855)	(1.241)
*Industry*	0.243**	0.0163	-0.0100
	(0.115)	(0.0156)	(0.0111)
*Lnagr*	-4.131**	0.232	0.388
	(1.736)	(0.314)	(0.369)
Year Fixed Effect	Yes	Yes	Yes
City Fixed Effect	Yes	Yes	Yes
Monitoring Point Fixed Effect	Yes	Yes	Yes
River System Fixed Effect	Yes	Yes	Yes
Constant	-59.38**	-8.301	5.835
	(29.32)	(6.581)	(9.671)
Obs	910	910	910
R^2^	0.700	0.808	0.703

Note: The robust standard errors are in parentheses;

*** means p <0.01,

** means p <0.05, and

* means p <0.1.

*chao×t* is a policy variable where chao is a dummy variable that is equal to 1 if the Chaohu Lake observation point is observed, and t is a dummy variable that is equal to 1 after the administrative division adjustment.

**Fig 4:**
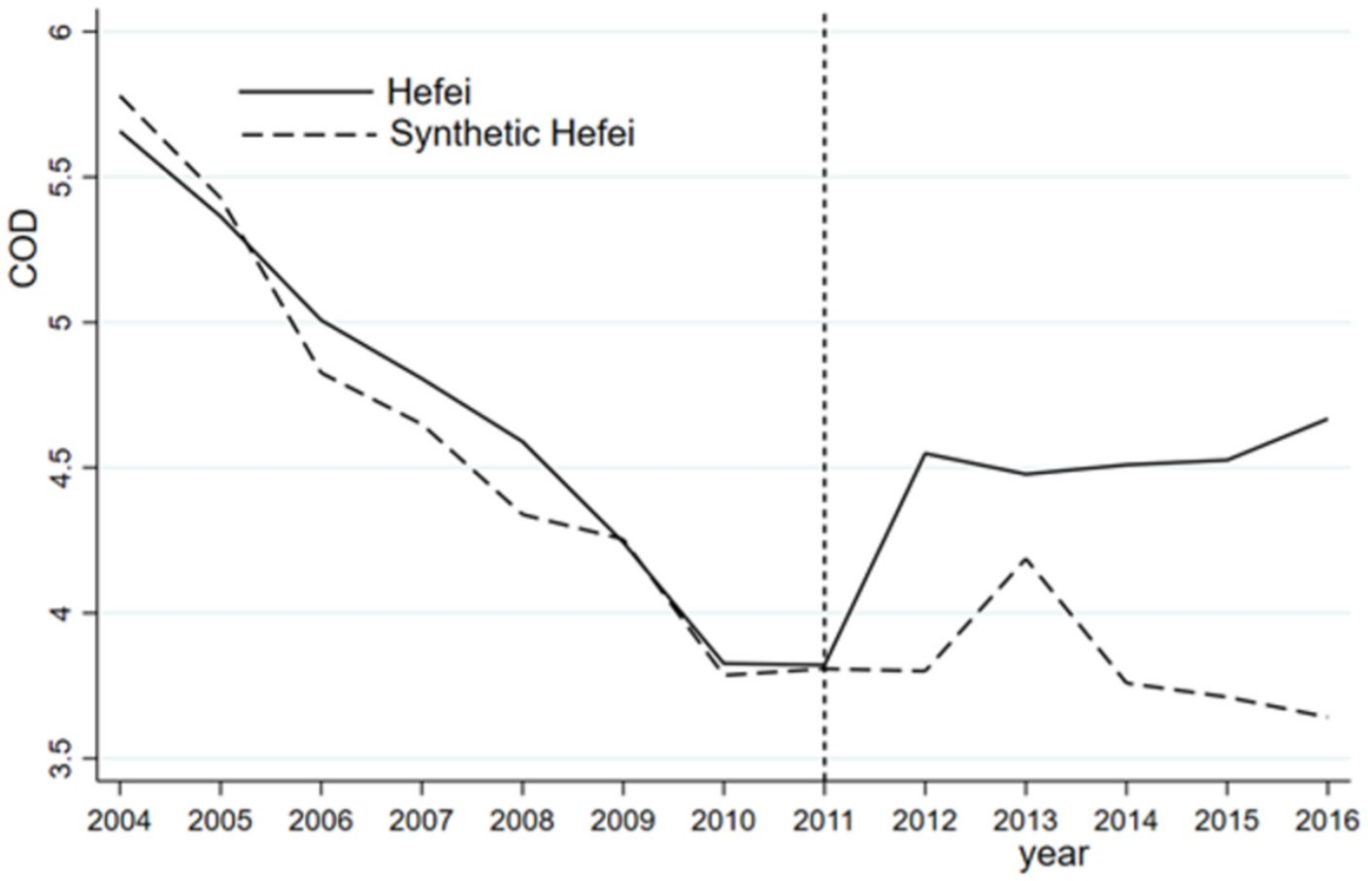
COD of Hefei and synthetic Hefei (excluding Beijing, Shanghai, and Tianjin).

**Fig 5:**
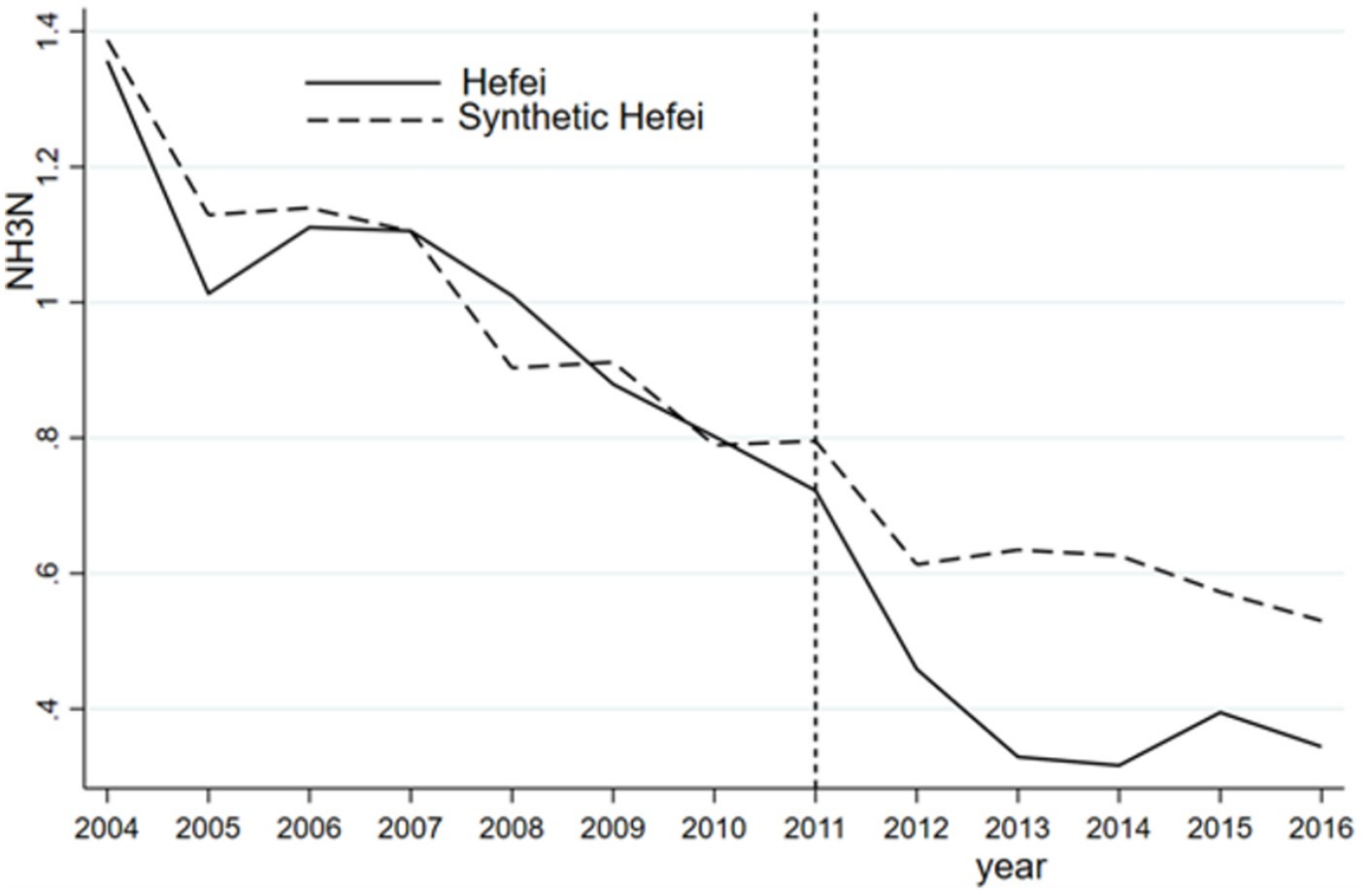
NH_3_N of Hefei and synthetic Hefei (excluding Beijing, Shanghai, and Tianjin).

**Fig 6:**
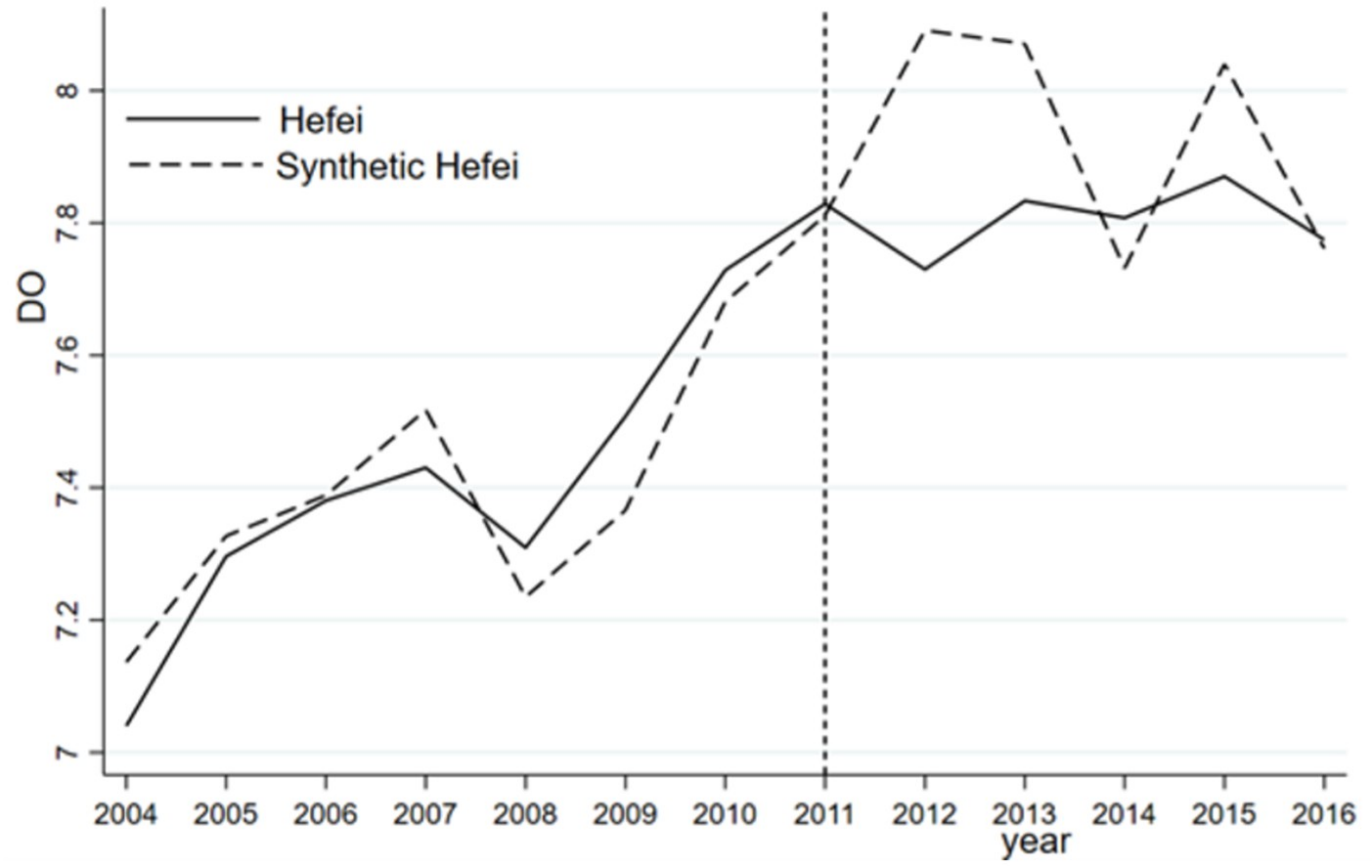
DO of Hefei and synthetic Hefei (excluding Beijing, Shanghai, and Tianjin).

**Fig 7:**
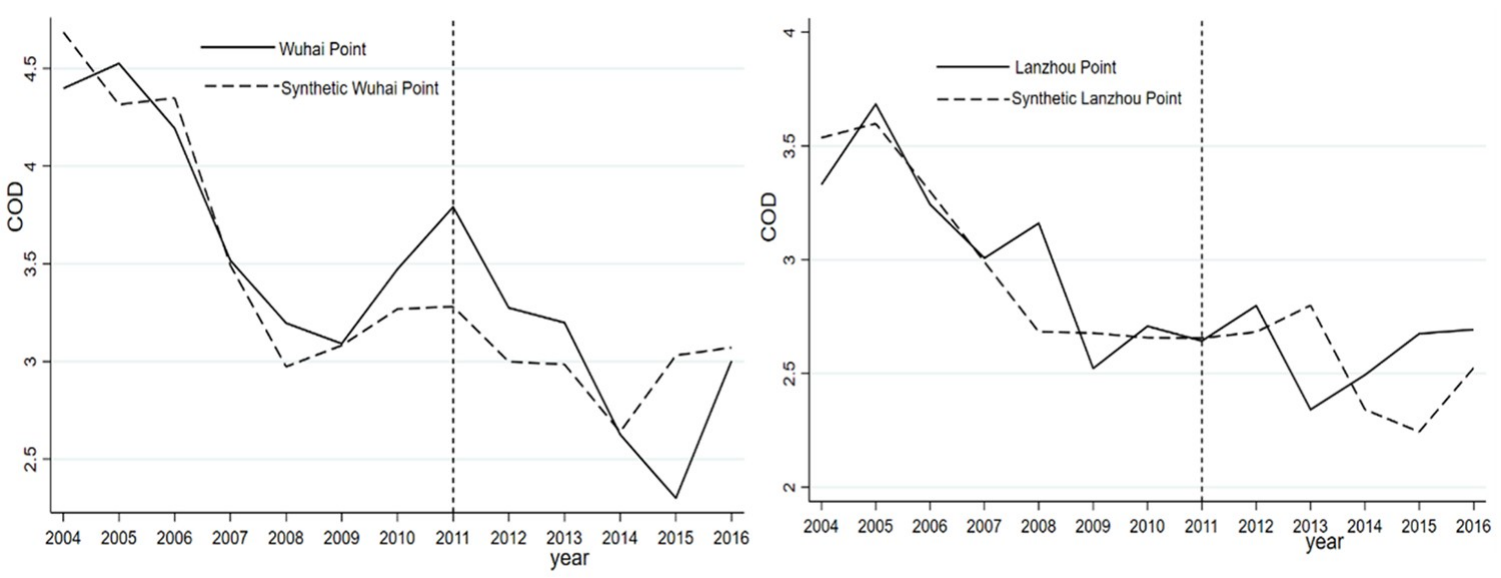
The synthetic effect of COD on the largest and smallest weights in the control group.

1. Prefecture-level cities are used in this study. The municipalities of Beijing, Shanghai, and Tianjin are significantly different from those in other provinces, such as Hefei, in terms of economic characteristics and other aspects. Therefore, the data for the three municipalities of Beijing, Shanghai, and Tianjin are excluded from the construction of the control group of synthetic Hefei for the three indicators. As shown in Table 7, the regression results confirm that the new synthetic Hefei and the previous synthetic Hefei are roughly similar. For instance, the administrative division adjustment improves the NH_3_N index but not the COD and DO indices (Figs 4–6).

2. From Table 7, we observe that the changes in COD and NH_3_N are significant at the 5% and 1% levels, respectively, while the change in the DO index is not significantly different from zero. However, the DID method is not appropriate for evaluating administrative division adjustment policies. The reason is that the DID method has three major problems when addressing the possible impacts of administrative division adjustments on water quality [44]. (a) The selection of the reference group is subjective and arbitrary. (b) The DID method requires the experimental group and control group to have the same trend before implementation of the policy, that is, the parallel trend assumption. However, the experimental group has only one observation, i.e., Hefei, and it is difficult to satisfy this assumption. (c) The problem is endogenous. In many cases, this endogeneity cannot be ruled out, and direct estimation using the DID approach will produce biased results. In contrast, our SCM is more robust to common factor shocks and nonlinearities than the DID method.

3. Following Abadie and Gardeazabal [36] and Abadie et al. [35], we conduct a placebo test for robustness purposes. Ideally, the study will identify a place without division adjustment and compare the water quality index between Hefei and synthetic Hefei. Since weights are important for synthetic variables, the two observation points with the largest and smallest weights in the control group (i.e., Xinchengqiao and Haibowan) are tested with a placebo to observe the changes in their water quality indicators. Fig 7 shows the observations with the largest and smallest weights in the synthetic control group. If a gap is observed, then the SCM has failed to provide sufficient evidence that the administrative division adjustment affects the water quality of Chaohu Lake. From Fig 7, there is no difference between the actual and synthetic water quality indices, indicating that there is no obvious policy impact on the synthetic effect of the Xinchengqiao and Haibowan Monitoring Stations.

4. The root mean square prediction error (RMSPE) test is also conducted for the periods before and after the intervention. The SCM eliminates the uncertainty of microlevel data in estimating the policy results. A placebo test, as proposed by Abadie et al. [35], is used to assess the robustness of the COD, NH_3_N, and DO indices. A total of 69 observation points in 2011 that are not involved in the administrative division adjustment are considered in this robustness test. One of the observation points is assumed to undergo an adjustment, and a synthetic version of this region is constructed using other regions outside Hefei. A regression model is applied to the three indicators, COD, NH_3_N, and DO. If the regression results are significant, then the difference is not caused by the division adjustment policy but by chance. A large RMSPE indicates that the fitting effect of the SCM may not be appropriate. The RMSPE before the intervention is expressed as follows:

RMSPEpre=1T0∑t=2004T0 y1t-∑j=2j+1 w*yjt2 (3)

Similarly, the RMSPE after the intervention takes a different time period for the average interval. There are several possible outcomes as follows. If the division adjustment policy has a significant effect on Hefei but not on synthetic Hefei, then the model cannot be predicted well and will lead to a larger postintervention RMSPE. As a result, a placebo effect exists only for synthetic Hefei. Moreover, if the results for synthetic Hefei are not well predicted before the intervention, then the preintervention RMSPE will lead to a larger postintervention RMSPE. The ratio of the two can be used to control the impact before the intervention. On the other hand, if the administrative division adjustment has a large policy effect and small placebo effects at other observation points, then the ratio of the postintervention RMSPE to the preintervention RMSPE should be significantly higher in Hefei than at other observation points. Table 8 shows the ratios of the postintervention RMSPE to the preintervention RMSPE for the three indicators at each observation point. As shown in Table 8, for the COD index, the RMSPE values before and after the intervention in Hefei are higher than those of the other 69 observation points, indicating that the effect of the administrative division adjustment on the COD index is significant. Similar results are observed for the NH_3_N and DO indices. Overall, there is a significant policy effect of the administrative division adjustment of Chaohu, and the SCM has a good fitting effect.

**Table 8:** Ratio of the RMSPE after and before the administrative division adjustment (post-RMSPE/pre-RMSPE).

ID	Province	City	COD	NH3N	DO	ID	Province	City	COD	NH3N	DO
1	Shanghai	Shanghai	0.6734	0.3690	2.9564	23	Shandong	Linyi	2.5305	0.1179	0.8819
2	Yunnan	Kunming	1.4919	0.2630	0.5278	24	Shandong	Zaozhuang	0.3179	0.5217	1.0110
3	Yunnan	Kunming	1.4922	0.2630	0.5331	25	Shandong	Jinan	2.8683	1.0434	1.0773
4	Inner Mongolia	Wuhai	0.8013	0.3600	1.8271	26	Shanxi	Yizhou	0.2973	0.6635	0.8535
5	Inner Mongolia	Baotou	0.5626	0.4909	2.0934	27	Shanxi	Yuncheng	0.2491	0.5905	0.6278
6	Beijing	Beijing	0.7124	0.3543	1.5660	28	Guangdong	Guangzhou	0.5819	0.8724	1.2096
7	Beijing	Beijing	0.7305	0.6873	1.9775	29	Guangdong	Qingyuan	1.2379	0.3010	1.1062
8	Jilin	Baicheng	2.5117	0.5763	0.4093	30	Guangxi	Pingxiang	0.8242	1.3118	1.1302
9	Jilin	Changchun	1.4313	0.7768	1.6882	31	Guangxi	Nanning	1.0335	0.5667	1.4244
10	Sichuan	Leshan	1.0634	0.8544	1.3291	32	Guangxi	Guilin	0.8370	0.6049	2.2648
11	Sichuan	Yibin	0.2963	0.5953	0.4286	33	Guangxi	Wuzhou	0.5575	0.2560	4.1551
12	Sichuan	Panzhihua	1.0919	0.6345	0.3912	34	Guangxi	Guigang	0.8683	0.5885	0.9247
13	Sichuan	Luzhou	0.4238	0.3856	0.8854	35	Jiangsu	Nangjing	0.5850	0.7046	1.5708
14	Tianjin	Tianjin	0.5771	1.8599	3.7492	36	Jiangsu	Yixing	1.8371	0.7966	1.3845
15	Tianjin	Tianjin	0.3517	0.8844	1.6014	37	Jiangsu	Yangzhou	0.5687	0.3569	0.4033
16	Anhui	Hefei	3.6169	1.7535	3.6136	38	Jiangsu	Wuxi	1.8371	0.7965	1.3842
17	Anhui	Anqing	0.5591	0.4670	1.2333	39	Jiangsu	Suqian	0.7012	0.3475	2.0027
18	Anhui	Huaibei	1.7651	0.5411	0.5501	40	Jiangsu	suzhou	0.7131	0.9520	0.7442
19	Anhui	Huainan	1.0810	0.2876	0.9103	41	Jiangsu	Xuzhou	0.6468	0.0219	2.2489
20	Anhui	Fuyang	0.7814	0.4880	1.5441	42	Jiangxi	Jiujiang	0.7882	0.2799	0.4785
21	Anhui	Bengbu	0.2824	0.5449	2.4805	43	Jiangxi	Jiujiang	0.8965	0.2419	0.4832
22	Anhui	Fuyang	0.7814	0.4880	1.5441	44	Jiangxi	Nanchang	0.6567	0.1337	3.3466
ID	Province	City	COD	NH3N	DO	ID	Province	City	COD	NH3N	DO
45	Hebei	Zhangjiakou	0.7341	0.1204	1.3325	58	Hunan	Changsha	0.6739	0.1806	0.8526
46	Hebei	Shijiazhuang	1.2374	0.7338	0.8283	59	Hunan	Changsha	0.6955	0.5080	1.7045
47	Hebei	Zhoukou	1.1023	0.2467	0.4273	60	Gansu	Lanzhou	1.4167	0.2004	1.2052
48	Hebei	Zhoukou	1.1023	0.2467	0.4273	61	Fujian	Fuzhou	0.6832	0.1025	2.7461
49	Hebei	Jiaozuo	0.8239	0.9146	0.8987	62	Liaoning	Fushun	0.3725	0.4147	0.3570
50	Hebei	Zhumadian	0.7342	0.0923	1.3735	63	Liaoning	Panjin	0.1954	0.2630	1.5290
51	Zhejiang	Jiaxing	0.5423	0.8051	0.8258	64	Liaoning	Yingkou	0.8573	0.6536	0.4338
52	Zhejiang	Hangzhou	0.4688	0.8188	0.9832	65	Liaoning	Liaoyang	0.3909	0.4917	3.0067
53	Zhejiang	Huzhou	0.1677	0.4330	0.6828	66	Liaoning	Tieling	0.2249	0.1566	0.9563
54	Hubei	Shiyan	0.6083	0.3080	0.8944	67	Chongqing	Chongqing	0.5374	0.6881	1.2156
55	Hubei	Yichang	1.2934	0.8323	0.6172	68	Heilongjiang	Tongjiang	0.3880	0.9562	1.6167
56	Hubei	Wuhan	0.2838	0.6456	1.2043	69	Heilongjiang	Zhaoyuan	0.2320	0.4491	0.8152
57	Hunan	Yueyang	0.7084	0.1993	0.9415	70	Heilongjiang	Heihe	1.0721	2.1523	1.0450

**Fig 8:**
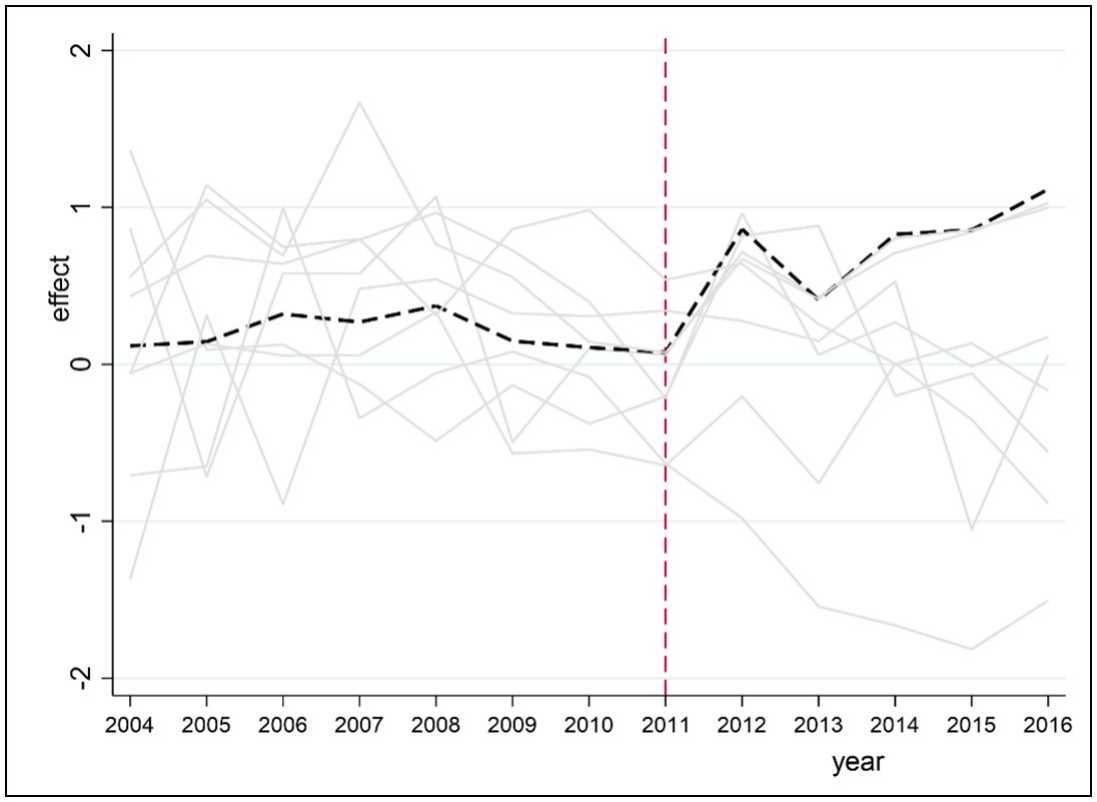
Prediction error distribution of COD for Hefei.

5. A placebo test is performed on each observation point for each indicator. If the observation point has a large RMSPE prior to the administrative division adjustment, then the observation point may not be suitable for constructing synthetic Hefei. Therefore, any observation point with an RMSPE greater than 3 times that of Hefei is removed. If values at some observation points are not well synthesized by the weighted averages at other observation points, then the difference between the actual and synthetic values may be due to the weighted average. Fig 8 shows the prediction error distribution of COD for Hefei and seven other observation points that meet the requirement. The black line represents the COD prediction error for Hefei, while the gray line represents the COD prediction error for the rest of the observation points. Before 2011, the prediction error for Hefei is moderate. After 2011, there is a significant difference between the true value and the composite value of COD in Hefei city. Only one observation point exceeds Hefei city in 2013, possibly due to the error caused by the weighted average. However, the results show that the administrative division adjustment has a significant negative effect on COD in Hefei.

**Fig 9:**
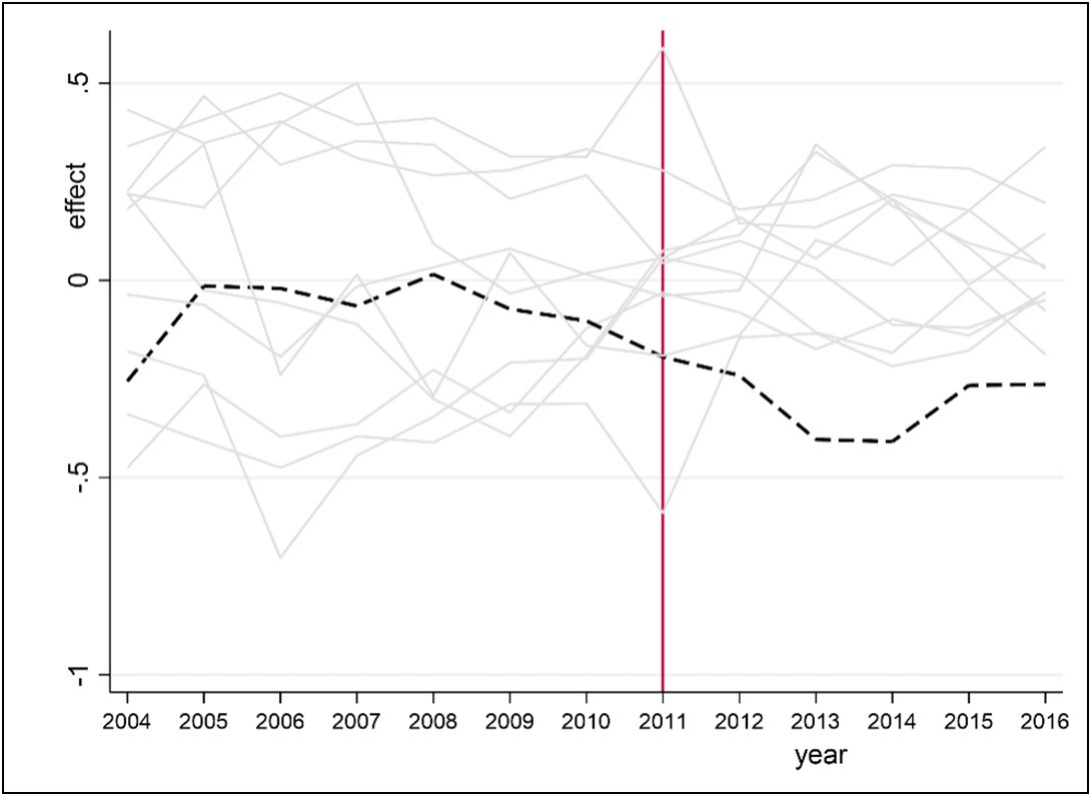
Prediction error distribution of NH_3_N for Hefei.

**Fig 10:**
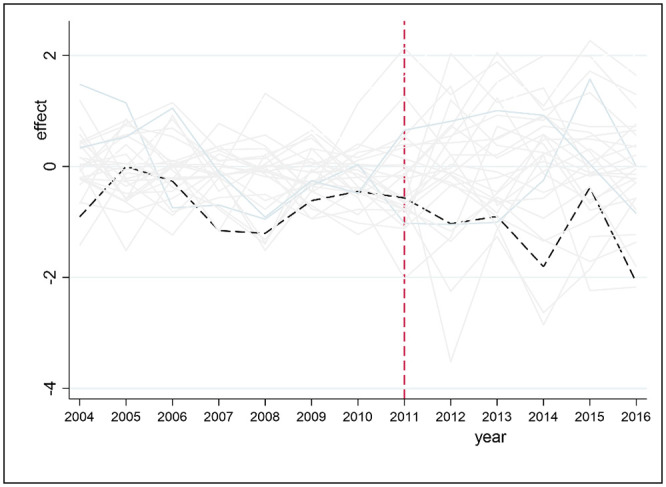
Prediction error distribution of DO for Hefei.

Fig 9 shows the prediction error distribution of the NH_3_N index between Hefei and the 10 other observation points. The black line represents the NH_3_N prediction error for Hefei, and the other gray lines represent the error distributions of the ten observation points. Overall, the gap between Hefei and the other observation points exhibits a downward trend from 2009 to 2016 and reveals that the administrative division adjustment has an impact on the ammonia nitrogen index of Hefei. In contrast, Fig 10 shows the placebo test results of the DO index in Hefei and 27 other observation points. The black dotted line represents the treatment effect on Hefei (the difference between the DO index of Hefei and that of synthetic Hefei), and the gray solid line represents the placebo effect on the 27 observation points (i.e., the difference between the DOs of these observations and their corresponding synthetic observations). Clearly, compared with the placebo effect on other observation points, the DO (negative) treatment effect on Hefei is larger, and the administrative division adjustment of Chaohu has a significant negative effect on the DO index of Hefei.

### 5.3 Economic and environmental effects of the administrative division adjustment of Chaohu

The economic and environmental impacts of the administrative division adjustment are further analyzed to explain the results.

#### 1. Impact of the administrative division adjustment of Chaohu on Hefei’s economy

A total of 284 prefecture-level cities are used for the synthesis. Table 9 shows that the coefficients of the variables in the synthesis group are similar to those in the experimental group, indicating that the synthesis effect is satisfactory.

**Table 9:** Comparison of the treated and synthetic predictors of Log (GDP).

Predictor Variables	Treated	Synthetic
Lnl	6.0493	5.9450
Lnfix	7.0365	7.0339
Second	47.3807	47.3840
Third	44.7195	44.7208
Labpercent	62.4605	56.4652
Lnpy	9.9563	9.9572
Lnarea	9.3474	9.3470
Lnpop	6.5209	6.5210

In Table 9, lny is the natural logarithm of the output of synthetic Hefei, lnl is the labor force, lnfix is fixed asset investment, second denotes the proportion of the secondary industry, third denotes the proportion of the tertiary industry, labpercent is the employment level, lnpy is the GDP per capita, lnarea is the city area, and lnpop is the urban population. The sample data are processed to ensure that the variables used for synthetic control are close to those of Hefei before the administrative division adjustment.

**Fig 11:**
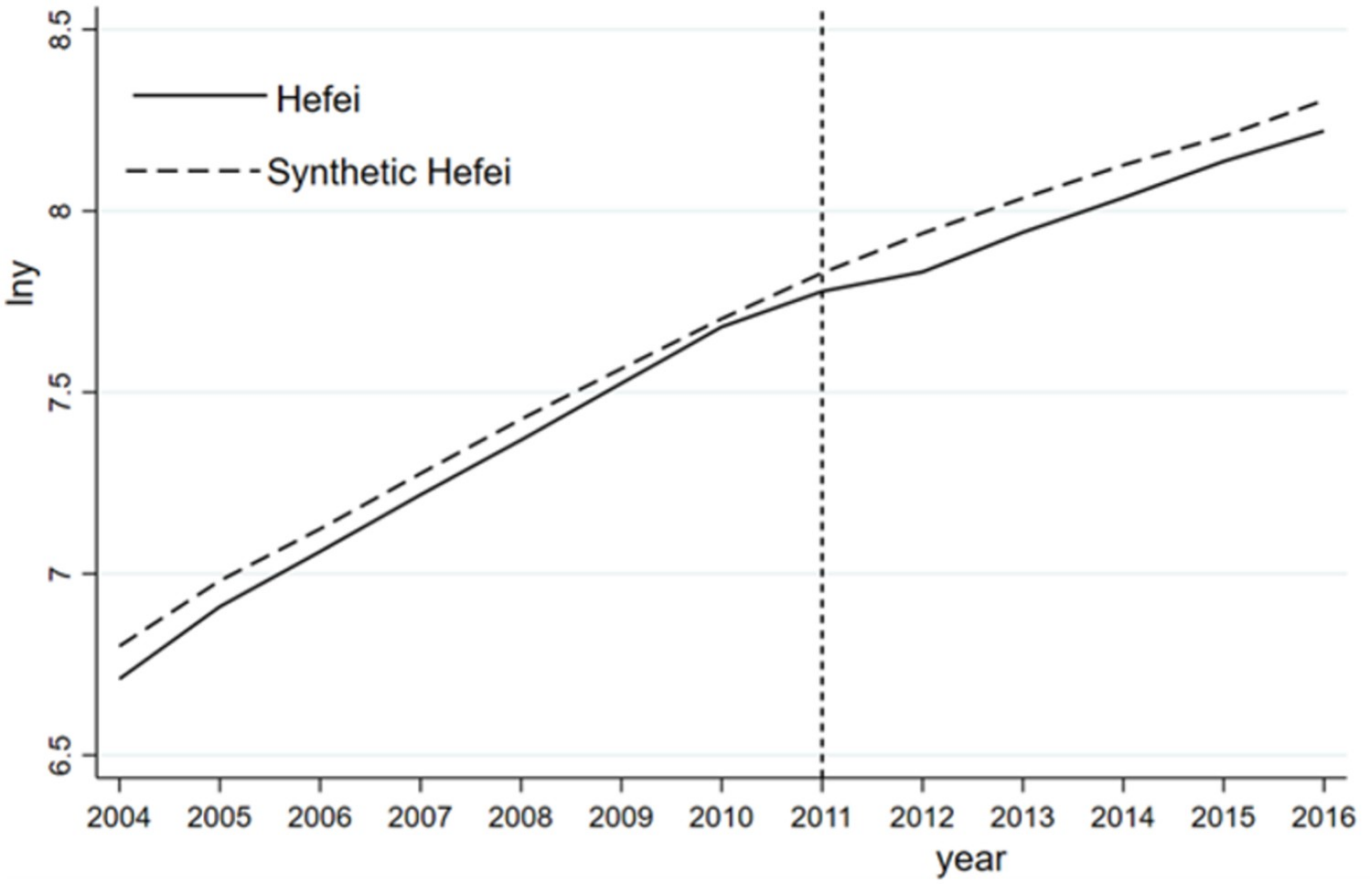
Log (GDP) of Hefei and synthetic Hefei.

The results indicate that the GDP of synthetic Hefei continues to grow after 2011. Fig 11 shows that Hefei’s GDP experiences a short-term downward trend in 2011 because the municipality of Chaohu is dominated by agriculture, because it has slow development and a large population, and because its overall economic level lags far behind that of Hefei city. After the abolition of the prefecture-level municipality of Chaohu and the abolition of Lujiang county as well as the merger with Chaohu city, the economic system of Hefei impacts the economy of Hefei. However, this change is not continuous, and there is a temporary decline in Hefei’s output.

**Fig 12:**
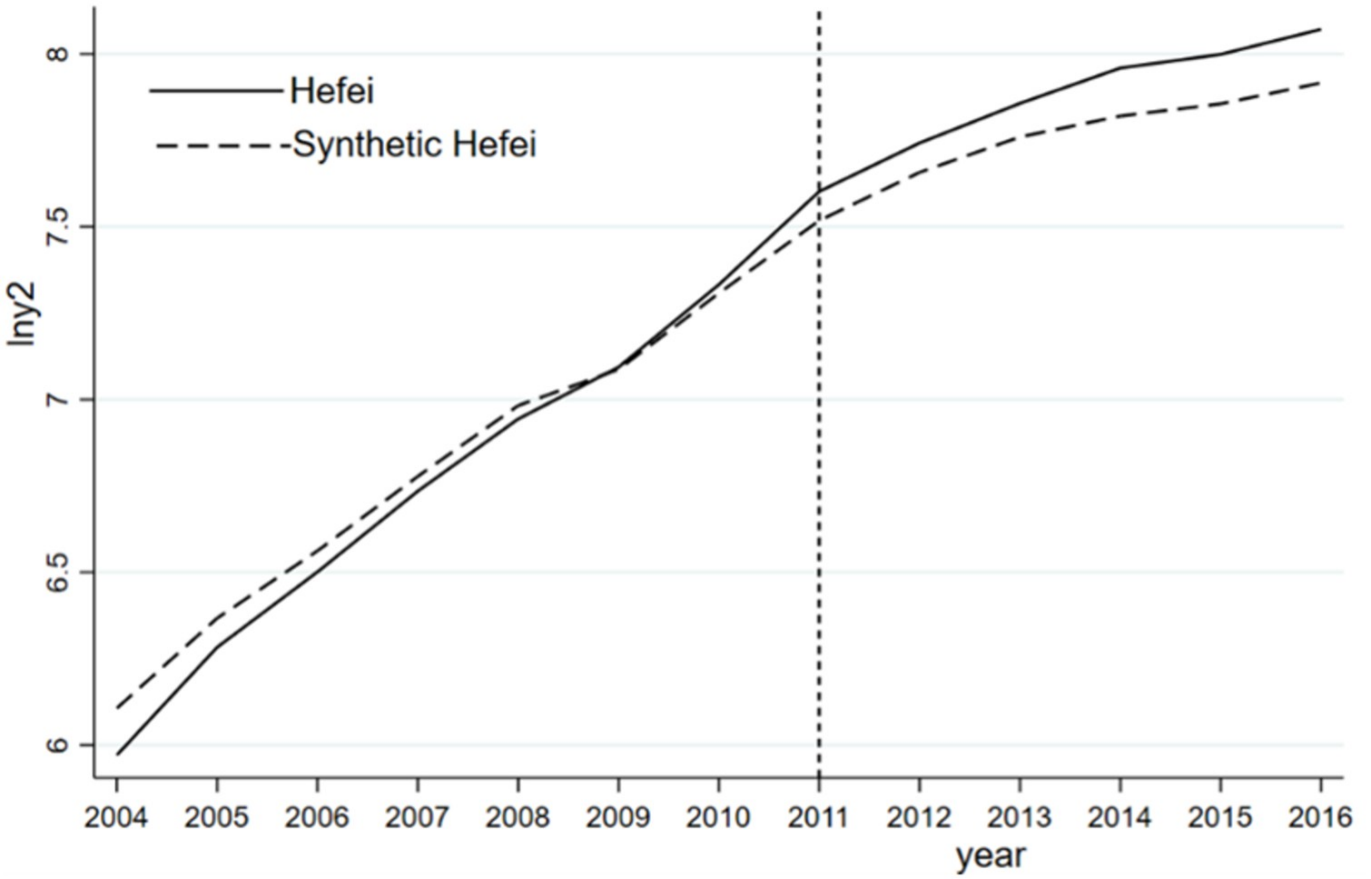
Log (GDP industry) of Hefei and synthetic Hefei.

To clearly illustrate the impact of the abolition of the prefecture-level municipality of Chaohu, a synthetic forecast of the GDP of the secondary industry is conducted. The synthetic results are shown in Table 10. Except for the proportion of the tertiary industry, the synthetic errors of the indicators indicate that the fitting effect is good and accurately simulates Hefei without the administrative division adjustment. In contrast, as shown in Fig 12, the trend of the GDP of the secondary industry contradicts the overall economic trend. The industrial development of Hefei surpasses that of synthetic Hefei, indicating that the administrative division adjustment has a significant driving effect on the industrial development of Hefei. Overall, the administrative division adjustment slows down the overall economic development rate of Hefei but improves its industrial growth, which is consistent with Hypothesis II. Regional growth and the governance of Chaohu Lake are more focused on economic growth, especially industrial expansion, weakening the governance of Chaohu Lake.

**Table 10:** Comparison of the treated and synthetic predictors of Log (GDP industry).

Predictor Variables	Treated	Synthetic
*Lnl*	6.0493	6.0368
*Lnfix*	7.0365	7.0101
*Second*	47.3807	47.3822
*Third*	44.7195	41.9444
*Labpercent*	62.4605	62.4595
*Lnpy*	9.9563	9.9564
*Lnarea*	9.3474	9.3469
*Lnpop*	6.5209	6.5220

#### 2. NH_3_N, COD, and DO indices

**Fig 13:**
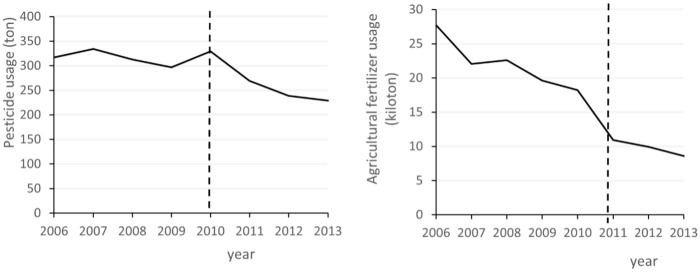
Pesticide and fertilizer consumptions in Hefei.

The decline in NH_3_N may be mainly due to industrial development replacing agricultural development. A series of industrial expansions, such as urban construction and real estate development, has caused a reduction in agricultural production. The use of chemical fertilizers and pesticides in Hefei shows a significant decline after the administrative division adjustment in 2011. Although the amount of agricultural fertilizer used by Hefei has shown a downward trend since 2006, the decline is even greater in 2011, reaching 40.12%. Fig 13 indicates that the amount of pesticide used is near 300 tons in the 2006–2010 period and then undergoes a large decline, reaching 18.23% in 2011, with a continued downward trend after 2011. The reduction in the scale of agriculture leads to a decline in the use of chemical fertilizers and pesticides. Therefore, the development of agricultural production is crowded out by industrial development, leading to a decline in the NH_3_N index.

The deterioration of the COD and DO indices is related to the rapid industrial and urban development. A large amount of domestic sewage and industrial sewage is discharged without effective treatment, resulting in an increase in the amount of water pollution in Chaohu Lake. Many sewage treatment plants have not been completed on time, leading to the direct discharge of domestic sewage. For instance, a low sewage treatment rate and the lack of treatment facilities have led to poor results. Industrial water intensity has been the main obstacle to the ecological security of the eastern half of Chaohu Lake in recent years, while the pollution in the western half of the lake mainly comes from urban development. The wetland area, domestic water intensity, and artificial forestation area are the main factors affecting the ecology of Chaohu Lake [45]. The deterioration of the COD and DO indices after the administrative division adjustment is closely related to the intensive industrial and urban development in Hefei.

There are several reasons for the poor environmental governance of Chaohu Lake after the administrative division adjustment, and they can be summarized as follows:

1. In terms of development space, urban construction has taken over ecological land, and a large number of illegal constructions have occurred. As stated by the central inspection team, as an expanded urban space, Hefei has seized a large amount of ecological land for commercial development, tourism development, and urban construction. Numerous commercial and real estate projects are illegally approved and constructed in the Chaohu Lake basin water environmental protection area. Moreover, there have been several problems in Chaohu; for instance, the wave-proof forest platform has been damaged, and aquatic plants have been completely destroyed, leading to the extinction of ecological functions. In the name of restoration, tourism development in Chaohu Lake continuously reduces wetlands and supports tourism development. The destruction of ecological wetlands reduces the self-recovery function and the recovery of the lake.
2. The poor management of Chaohu Lake is another major problem. A specialized organization for the management of Chaohu Lake was established in 2012 to provide unified protection and supervision. However, the Chaohu Administration Bureau is not meeting its environmental protection responsibilities, partly because of the mismatch between financial power and administrative power, for instance, between lake invasion and wetland destruction prevention.
3. The local government emphasizes the economy instead over the environment, and as a result, environmental regulations and pollution control projects are not implemented on schedule. For example, the Anhui Provincial Government adjusted the weights of target management assessments in prefecture-level cities in 2016. The weight of economic development rose from 14.6%-22.3% in the previous year to 27.5%-32.5%. However, the evaluation weight of ecological and environmental indicators changes from 14.6%-22.3% to 13.5%-20.5%. This deviation has led to economic development but poor environmental protection. Some government agencies are more inclined to engage in economic development and ignore environmental protection issues. For instance, the requirements stipulated in the "Chaohu Basin Water Pollution Prevention Regulations" have not been implemented, and the third phase of the Shiwili River Sewage Treatment Plant, initiated in 2013, has not been completed, resulting in approximately 60,000 tons of sewage being discharged into Chaohu Lake. Water pollution treatment in Chaohu Lake, which will affect sustainable economic development, increase the pressure on the environment, and lead to an improvement in water quality, is usually placed after economic development in terms of importance.
4. The limited role of nonprofit organizations and public participation explains the governance of Chaohu Lake. Learning from the experience of large lakes in Western countries, we point out that active public participation is required to obtain good lake water pollution treatment results. The public participates in pollution control in a variety of ways, such as by participating in the drafting of water pollution control policies and building their its awareness of ecological environment protection. Moreover, Lake Biwa in Japan is another good example of the important role played by nonprofit organizations and the public in water pollution prevention and control. Although the government always plays a dominant role in the treatment of lake pollution, the government alone is not enough to overcome the problem of water pollution. Collaboration between enterprises, nonprofit organizations, the public, and governmental bodies is required to establish a reasonable governance cooperation mechanism for managing Chaohu Lake.

## 6. Conclusions

Administrative division adjustments have various social, economic, and environmental effects. The previous literature has focused on the economic effects arising from administrative division adjustments and has ignored the environmental effects. The administrative division adjustment of Chaohu is conducive to environmental governance in the long term. The SCM is used to identify the effect of the administrative division adjustment on the water quality of Chaohu Lake. After the division adjustment, some water quality indicators, such as NH_3_N, improve, dropping by approximately 30%. However, other major pollution indicators, such as COD and DO, worsen to varying degrees. For instance, COD has increased by approximately 25%. After excluding municipalities from the sample, we compare the RMSPE before and after the administrative division adjustment. A placebo test applied to assess the robustness of the results indicates that the environmental policy effect of the administrative division adjustment is not satisfactory. Furthermore, several factors have influenced economic and industrial expansion, including the rapid expansion of urban areas, the imperfect management system for Chaohu Lake, and the emphasis on the economic performance of officials.

Several important policy implications can be drawn from the current study. First, returning to the initial intention of the administrative division adjustment, the main reason the prefecture-level municipality of Chaohu was split and merged was to improve the governance of Chaohu Lake. Prior to 2011, Chaohu Lake had the most unsatisfactory governance of the five largest freshwater lakes in China. The central government was determined to improve the management system for Chaohu Lake by dismantling and merging Chaohu city to achieve better environmental governance and economic development. Therefore, we re-emphasize and rationalize the governance mechanism to achieve the policy objectives of the administrative division adjustment plan. Measures should be taken to achieve this objective. Governments should make full use of modern monitoring methods such as drones, vigorously promote the modernization, automation, and informatization of lake monitoring, and continuously improve their monitoring, monitoring capabilities and monitoring efficiency. In addition, governments should establish and improve a comprehensive lake evaluation system and regularly evaluate the health and ecological safety of lakes. Furthermore, governments should accelerate improving the legal system for lake protection and management, promote joint law enforcement, and focus on improving the comprehensive supervision system. Moreover, financial support should be strengthened. Based on the division of fiscal powers and expenditure responsibilities, governments at all levels should reasonably arrange financial funds for lake protection and treatment and include eligible lake ecological environmental protection restoration projects in the scope of special bond support for local governments. Furthermore, governments should actively promote the establishment of a diversified investment and financing mechanism with government guidance, market operation, and social participation and guide state-owned enterprises, various financial institutions, and social capital to participate in lake protection and governance in accordance with laws and regulations. Governments should also regularly evaluate the effectiveness of lake protection and management and provide commendations and rewards in accordance with regulations to further highlight the demonstration role. Second, the focus should be shifted from economic development to high-quality development. To achieve this goal, we must not habitually rely on economic growth and environmental governance; instead, the government should promote economic transformation, develop strategic emerging industries, and cultivate industries such as biotechnology, new energy, new materials, and green environmental protection based on local conditions. In addition, it is necessary to clarify the power and responsibility for environmental monitoring in the Chaohu Lake basin. For instance, the environmental monitoring and enforcement functions should be the responsibility of the Chaohu Administration Bureau.

## Supporting information

S1 Data (DTA)
